# Standardization of Opiate Substitution Therapy in Brent, London

**DOI:** 10.1192/bjo.2026.11354

**Published:** 2026-06-30

**Authors:** Isha Bhat, Katie James

**Affiliations:** 1 Betsi Cadwaladr University Health Board, Llanfairfechan, United Kingdom; 2 CNWL NHS Foundation Trust, London, United Kingdom

## Abstract

**Aims:**

The aim of this Quality Improvement (QI) project was to standardize the use of Opiate substitution therapy (OST) in Brent, London to improve patient experience, streamline supervision level and reduce money spent on supervision.

Patients were initially provided with supervised prescription of OST with aim to progress to lower supervision as they demonstrate adherence and stability. There were some inconsistencies in supervision level leading to certain patients remaining on unnecessary high levels of supervision. Pharmacy time and resources were diverted to supervise patients who no longer required this level of monitoring. A structured questionnaire with guidance in accordance with Orange guidelines and Trust Policy was developed as part of a QI Project to streamline the process.

**Methods:**

The QI Project ran from October 2024 to August 2025 (8 months) and employed a Plan–Do–Study–Act(PDSA) approach and completed one PDSA Cycle.

Interventions included development of unified OST Supervision Questionnaire (countersigned by a prescriber), staff training and presentation about guidelines to reduce the supervision. Data on patient satisfaction, supervision cost and number of patients and level of supervision was collected quarterly.

**Results:**

Implementation of standardized OST Protocol resulted in a measurable increase in patient satisfaction. However, the project also identified an increase in pharmacy costs, specifically related to supervision. This rise was attributed to enrolment of a large cohort of new patients during April and May which temporarily increased supervision needs.

Baseline data showed 44.8% patients on supervised dose of OST (buprenorphine & methadone) which increased to 45.7% in March and further to 47.3% in July. This increase was mainly seen due to new clients enrolling and as part of new Guidance–patients needed to be on 2 months supervised dose before any reduction to this regimen could be made.

**Conclusion:**

The QI Project ran for 8 months successfully standardizing the approach to OST supervision in Brent, London. It led to enhanced patient satisfaction and increase in follow-up by patients. Though supervision cost and number of patients on supervised doses increased by small percentage which was contributed to the surge of new patient enrolment. The questionnaire provided a great therapeutic tool to key workers to engage in an open conversation around supervision level. The overall outcome suggested that standardizing OST Process was beneficial and sustainable with regular staff training and case discussion with line managers.

